# Why SNP rs227584 is associated with human BMD and fracture risk? A molecular and cellular study in bone cells

**DOI:** 10.1111/jcmm.13991

**Published:** 2018-10-28

**Authors:** Xu Zhou, Ying‐Hua Qiu, Pei He, Fei Jiang, Long‐Fei Wu, Xin Lu, Shu‐Feng Lei, Fei‐Yan Deng

**Affiliations:** ^1^ Center for Genetic Epidemiology and Genomics School of Public Health Soochow University Suzhou Jiangsu China; ^2^ Jiangsu Key Laboratory of Preventive and Translational Medicine for Geriatric Diseases School of Public Health Soochow University Suzhou Jiangsu China

**Keywords:** *C17orf53*, osteoblast, osteoporosis, phosphorylation, SNP

## Abstract

A large number of SNPs significant for osteoporosis (OP) had been identified by genome‐wide association studies. However, the underlying association mechanisms were largely unknown. From the perspective of protein phosphorylation, gene expression regulation, and bone cell activity, this study aims to illustrate association mechanisms for representative SNPs of interest.

We utilized public databases and bioinformatics tool to identify OP‐associated SNPs which potentially influence protein phosphorylation (phosSNPs). Associations with hip/spine BMD, as well as fracture risk, in human populations for one significant phosSNP, that is, rs227584 (major/minor allele: C/A, EAS population) located in *C17orf53* gene, were suggested in prior meta‐analyses. Specifically, carriers of allele C had significant higher BMD and lower risk of low‐trauma fractures than carriers of A. We pursued to test the molecular and cellular functions of rs227584 in bone through osteoblastic cell culture and multiple assays.

We identified five phosSNPs significant for OP (*P *< 0.01). The osteoblastic cells, which was transfected with wild‐type *C17orf53* (allele C at rs227584, P126), demonstrated specific interaction with *NEK2* kinase, increased expression levels of osteoblastic genes significantly (*OPN, OCN, COL1A1*,* P *< 0.05), and promoted osteoblast growth and *ALP* activity, in contrast to those transfected with mutant *C17orf53* (allele A at rs227584, T126).

In the light of the consistent evidences between the present functional study in human bone cells and the prior association studies in human populations, we conclude that the SNP rs227584, via altering protein‐kinase interaction, regulates osteoblastic gene expression, influences osteoblast growth and activity, hence to affect BMD and fracture risk in humans.

## 1 INTRODUCTION

OP is a complex chronic bone disease characterized by reduced bone mass, resulting in microarchitectural deterioration of bone tissue and increased bone fragility.^1^ Osteoporotic fracture (OF), the most serious consequence of OP, has high disability and mortality. In China, the prevalence of OP increases from 14.94% before 2008 to 27.96% during 2012‐2015, which places an enormous economic burden on the whole society.^2^

Bone mineral density (BMD) is a classical diagnosis standard for OP and has high heritability.^3^ Over the past decades, plentiful genetic and genomic studies have identified genes that are associated with BMD variation in human populations. However, the underlying molecular and cellular mechanisms for most of the established associations were largely unknown, leaving the molecular pathophysiology of OP/OF unclear yet.

Single‐nucleotide polymorphisms (SNPs) make up approximately 90% of human genetic variations.^4^ Nonsynonymous SNPs (nsSNPs) account for nearly half of the genetic variations associated with inherited diseases.^5^ Notably, almost 70% of nsSNPs in human genome are potential phosphorylation‐related SNPs (abbreviated as phosSNPs).^6^ Protein phosphorylation is a reversible posttranslational modification, which plays critical roles in various signalling pathways,^7^ being involved in transcription factor activation and gene expression,^8^, ^9^ and regulates essential cellular processes, for example, metabolism, differentiation, and membrane transportation.^10^, ^11^, ^12^ In eukaryotes, different protein kinases (PKs) could specifically recognize and attach phosphate groups to target amino acid residues serine (S), threonine (T), and tyrosine (Y).^13^ Previous studies have manifested that abnormal regulation of protein phosphorylation was related to pathogenesis of various diseases, like cancer, diabetes, osteoporosis, and so on.^14^, ^15^, ^16^

From the perspective of protein phosphorylation, gene expression, and cellular functions, the present study aims to ascertain the association mechanisms for significant OP‐related SNPs and OP. Through searching public resources for SNPs significant for OP, and predicting the potential impacts of filtered SNPs on protein phosphorylation, we identified significant phosSNPs influencing OP. Based on the hints from the bioinformatics prediction and previous GWAS studies, we focused our investigation on a representative SNP of interest, that is, phosSNP rs227584, and carried out purposeful downstream assays in vitro to ascertain its molecular and cellular functions.

Evidences, collected from the present molecular and cellular study in bone cells, support that SNP rs227584 changes protein phosphorylation site, regulates osteoblastic gene expression, and influences osteoblast functions. Our findings filled the gap of knowledge and illustrated the mechanism underlying the association between rs227584 and BMD/fracture risk in human populations. The present study highlights the significance of rs227584 and protein phosphorylation in bone biology, and shed new insights into the molecular pathogenesis of OP in humans.

## 2 MATERIALS AND METHODS

### 2.1 Searching database for OP‐associated phosSNPs

We searched public resources (dbGap, NHGRI) in Phenotype‐Genotype Integrator (available at https://www.ncbi.nlm.nih.gov/gap/phegeni) for SNPs significant for OP, including traits of interest “bone density,” “osteoporosis,” and “fracture.” All the picked SNPs are located in the exon, neargene, and UTR regions. From the list of OP‐associated SNPs (*P *< 0.01), phosSNPs were extracted for follow‐up molecular and cellular functional studies.^6^

### 2.2 Predicting and validating impacts of SNP rs227584 on protein functions

GPS2.0 software was utilized to predict the potential impacts of phosSNPs on protein phosphorylation.^17^ The phosSNP of interest, that is, rs227584, is located in *C17orf53* gene (Chromosome 17 open reading frame 53) on 17q21. Based on the bioinformatics prediction results, we carried out the following experiments to validate its impacts on protein molecular functions in osteoblastic cells, including change in protein substrate‐kinase interaction and change in *C17orf53* total protein phosphorylation. The procedures for the above experiments are detailed as follows.

#### 2.2.1 MG63 cell culture

Human osteoblastic‐like cell line MG63 was purchased from the Institute of Cell Bank/Institutes for Biological Sciences (Shanghai, China, http://www.cellbank.org.cn). MG63 was a kind of human osteosarcoma cells, which shares many similar features to undifferentiated osteoprogenitors, including a high proliferative capacity and similar expression profiles of many osteoblastic markers such as *ALP, OCN, OPN,* and *COL1A1*.^18^, ^19^ In this study, MG63 cells were cultured in DMEM (HyClone, Catalogue No.SH30022.01) supplemented with 10% foetal bovine serum (FBS, Gibco, Catalogue No.A31608‐01) and incubated at 37°C in a 5% CO_2_ atmosphere. Penicillin (100U/mL) and streptomycin (100U/mL) were added in the medium.

#### 2.2.2 MG63 cell transient transfection

Three allele‐specific *C17orf53* (T120P126, T120T126, A120P126) cDNA sequences were cloned into the target region of vector plasmid (Figure S1A), respectively, generating three novel plasmids containing various alleles at *C17orf53* gene (wild‐type: pCMV6‐*C17orf53*‐T120**P126**, mutant: pCMV6‐*C17orf53*‐T120**T126**, and variant: pCMV6‐*C17orf53*‐A120P126). The wild‐type cDNA sequence was generated from the *C17orf53* transcript variant 1 (NCBI Reference sequence: NM_024032.4).The pCMV‐Entry Vector plasmid was employed as negative control. The variant pCMV6‐*C17orf53*‐A120P126 was designed to substitute the suspect target site of phosphorylation (Threonine at amino acid residue 120, T120) with an unphosphorylatable residue (Alanine, A120) at the *C17orf53* protein product.

The MG63 cells were seeded at approximately 3 × 10^5^ cells/well in 6‐well plates. After 24 h, cells were transfected with 2.0 μg of plasmids, 4.0 μL of Lipofectamine^®^ 3000 reagent, 4.0 μl P3000™ reagent, and 125 μl Opti‐MEM ^®^ Medium per well (Life Technologies, Catalogue No.L3000‐015). After 72 h, MG63 cells were harvested and lysed for downstream assays. All transfection experiments were conducted in duplicate for each plasmid and repeated for three times.

Then, the transiently transfected MG63 cells were prepared for experimental validation of phosSNP rs227584 on protein functions, as follows.

#### 2.2.3 C17orf53‐NEK2 protein interaction assay

Seventy‐two hours after transient transfection, MG63 cells were lysed on the ice with cell lysis buffer (Beyotime, Order No. P0013), and then, the total proteins were collected by centrifugation (14 000 × *g*, 10 min). The protein concentration was measured with a BCA Protein Assay Kit (Beyotime, Order No.P0012S).

For the acquired supernatant proteins, co‐immunoprecipitation (Co‐IP) followed by Western blotting procedures were utilized to test substrate‐kinase interaction between *C17orf53* protein and the predicted kinase *NEK2*. Specifically, the supernatants of cell lysate were incubated with mouse anti‐human *NEK2* monoclonal antibody (Santa Cruz Biotechnology, Catalogue No.sc‐55601) and Protein A+G Agarose (Beyotime, Order No.P2012) overnight at 4°C. After washing for five times and pelleting at 2500 rpm/min, 5 min, the precipitate was resuspended in approximately 50 μL cell lysis buffer at final. Mouse IgG (Beyotime, Order No.A7028) was utilized as negative control of the Co‐IP experiment. The Co‐IP product was separated by electrophoresis on 8% SDS‐PAGE gel and then transferred to PVDF membrane. The membrane was firstly incubated with mouse‐anti‐human *C17orf53* monoclonal antibody (LifeSpan BioSciences, Catalogue No.LS‐C191789/52739), and then incubated with goat anti‐mouse HRP‐conjugated secondary antibody (CMCTAG, Catalogue No.AT0098). Protein bands were visualized by BeyoECL Plus (Beyotime, Order No.P0018) and imaged with GeneSys software (SYNGENE, GBOX chemi XL1.4). The above substrate‐kinase interaction assays were repeated twice.

#### 2.2.4 C17orf53 protein phosphorylation assay

Seventy‐two hours after transient transfection, we collected MG63 cell lysate and purified *C17orf53* protein with anti‐DDK antibody to pull down *C17orf53* protein, which had been tagged by the DDK flag in the constructed plasmid (Figure S1A). Then, *C17orf53*‐specific protein phosphorylation level was quantified using a Phosphate Assay Kit (Abcam, Catalogue No.ab65622) according to manufacturer's instructions. The assay utilized a proprietary formulation of malachite green and ammonium molybdate which forms a chromogenic complex with phosphate ion, giving an intense absorption band around 650 nm. Microplate Reader (BioTek, Synergy 2) was used to measure and collect the data. Protein Phosphorylation Assays were performed in duplicate for each condition and repeated three times.

### 2.3 Testing effects of phosSNP rs227584 on osteoblastogenesis in vitro

#### 2.3.1 MG63 cell stable transfection

Two rs227584 allele‐specific *C17orf53* (P126 andT126) cDNA sequences were cloned into the “Gene Insert” region of lentivirus expression plasmid pLenti‐GIII‐CMV‐GFP‐2A‐puro (Figure S1B) with Tranzyme cloning kit (Applied Biological Materials Inc. Cat No.E044) to generate plasmids carrying wild‐type and mutant *C17orf53*, respectively. The vector plasmid in Figure S1B was employed as negative control. The vector and the two newly constructed plasmids, together with 2nd Generation Packaging Mix (Applied Biological Materials Inc. Cat No.LV003), were coinfected into 293T cells with Lentifectin™ Transfection Reagent (Applied Biological Materials Inc. Cat No.G074) according to the manufacturer's instructions, generating wild‐type (*C17orf53*‐P126) and mutant (*C17orf53*‐T126) vector lentivirus. After 48 h, lentivirus was harvested and purified by filtering through 0.45‐μm low protein binding PES filter membrane. The concentrated lentivirus was obtained after high‐speed centrifuging for 4 h at 10 000 × *g*.

MG63 cells were seeded at 24‐well plates with 20%‐35% cell density per well. After 24 h, the prepared lentivirus was added to coculture with MG63 cells. After 36 h culture, a series concentrations of puromycin (0.4, 0.6, 0.8, 1.0, 1.2 μg/mL) were added to the culture medium to determine the appropriate concentration. After 1 week selection, 1.0 μg/mL puromycin was maintained in the stable cell culture.

Then, the stably transfected MG63 cells were used for testing effects of phosSNP rs227584 on osteoblastogenesis in vitro. To determine the infection efficiency towards stable transfection, MG63 cells expressing GFP marker protein were imaged using fluorescence microscope (BIO‐RAD, ZOE fluorescent cell imager, America) 48 h after infection.

#### 2.3.2 Cell growth assay

Stably transfected MG63 cells were seeded at 5 × 10^3^/well in 96‐well plates. The experiment was conducted in quadruplicate for each condition of cell. According to the preset time interval, MG63 growth process was monitored and recorded in real time by the RTCA S16 instrument (Model: 1×16, Serial No: 58‐1‐1509‐1086‐4, China) according to the manufacturer's instructions. The 24‐hour dynamic growth trends were exported as time‐dependent graph.

#### 2.3.3 Quantitative real‐time PCR

Osteopontin (*OPN*) and alkaline phosphatase (*ALP*) had been recognized to be involved in the osteoblastic differentiation process and are commonly used as “osteoblastic indexes.” In bone tissue, collagen 1 (*COL1A1* and *COL1A2*) constitutes the fundamental structural framework for bone formation and mineral deposition, and the collagen fibre gives tensile strength to bone. Osteocalcin (*OCN*), also known as bone gamma‐carboxyglutamic acid‐containing protein, is a noncollagenous matrix protein in bone, which is involved in the bone matrix maturation and ordered deposition of hydroxyapatite.

To evaluate the molecular effect of rs227584 on osteoblastogenesis and bone formation, we compared mRNA expression levels (*OPN, COL1A1,* and *OCN*) and enzyme activity (*ALP*) of MG63 cells that had been stably transfected with wild‐type or mutant *C17orf53* gene. The experimental procedures are described as followed.

MG63 cells, transfected stably with vector, wild‐type, or mutant *C17orf53*, were seeded at approximately 3 × 10^5^ cells/well in 6‐well plates. After 72 h, the cells were lysed and reverse transcribed with GoScript™ Reverse Transcriptase (Promega, Catalogue No.A5003) according to the manufacturer's instructions, respectively. The mRNA expression levels of *OPN, COL1A1*, and *OCN* genes were quantified by quantitative real‐time PCR (Life Technologies, QuantStudio 6 Flex). The mRNA expression levels were normalized against that of Glyceraldehyde 3‐Phosphate Dehydrogenase (*GAPDH*, internal control). The vector transfection was used as negative control of the experiment. The PCR primers used are presented in Table S1. The quantitative RT‐PCR experiments were performed in triplicates for each condition and repeated three times.

#### 2.3.4 ALP staining and osteoblast activity assay

Stably transfected MG63 cells were seeded at 2.15 × 10^4^/well in 24‐well plates and incubated at 37°C in 5% CO_2_ for 7 days. On day 8, *ALP* enzyme activity was assessed by TRACP & ALP Double‐Stain Kit (Takara, Catalogue No. MK300) following the manufacturer's instructions. The experiments were performed in triplicate for each condition and repeated for three times.

### 2.4 Inquiring eQTL effect of rs227584 in phenotype‐genotype integrator

A study in a large cohort showed that about 48% of previously published significant GWAS SNPs are significant eQTLs in human whole blood cells.^20^ Based on the public resources Phenotype‐Genotype Integrator (available at https://www.ncbi.nlm.nih.gov/gap/phegeni), we entered SNP rs number 227584 under the Genotype Selection and searched for its potential eQTL effects in all human tissues if available.

## 3 RESULTS

### 3.1 Identification of phosSNPs significant for OP

Based on the findings from published genome‐wide association studies and archived in public resources, five phosSNPs significant for OP were identified (Table S2). Most interesting, associations with hip/spine BMD as well as fracture for one of the phosSNPs, that is, SNP rs227584 (major/minor allele: C/A, EAS population) located in *C17orf53* gene, were suggested in prior meta‐analyses in large human populations of European and East Asian ancestry. Specifically, allele C carriers have significant higher hip and spine BMD and lower low‐trauma fracture risk than allele A carriers.^21^

### 3.2 Predictive impacts of phosSNP rs227584 on protein functions

**Figure 1:**
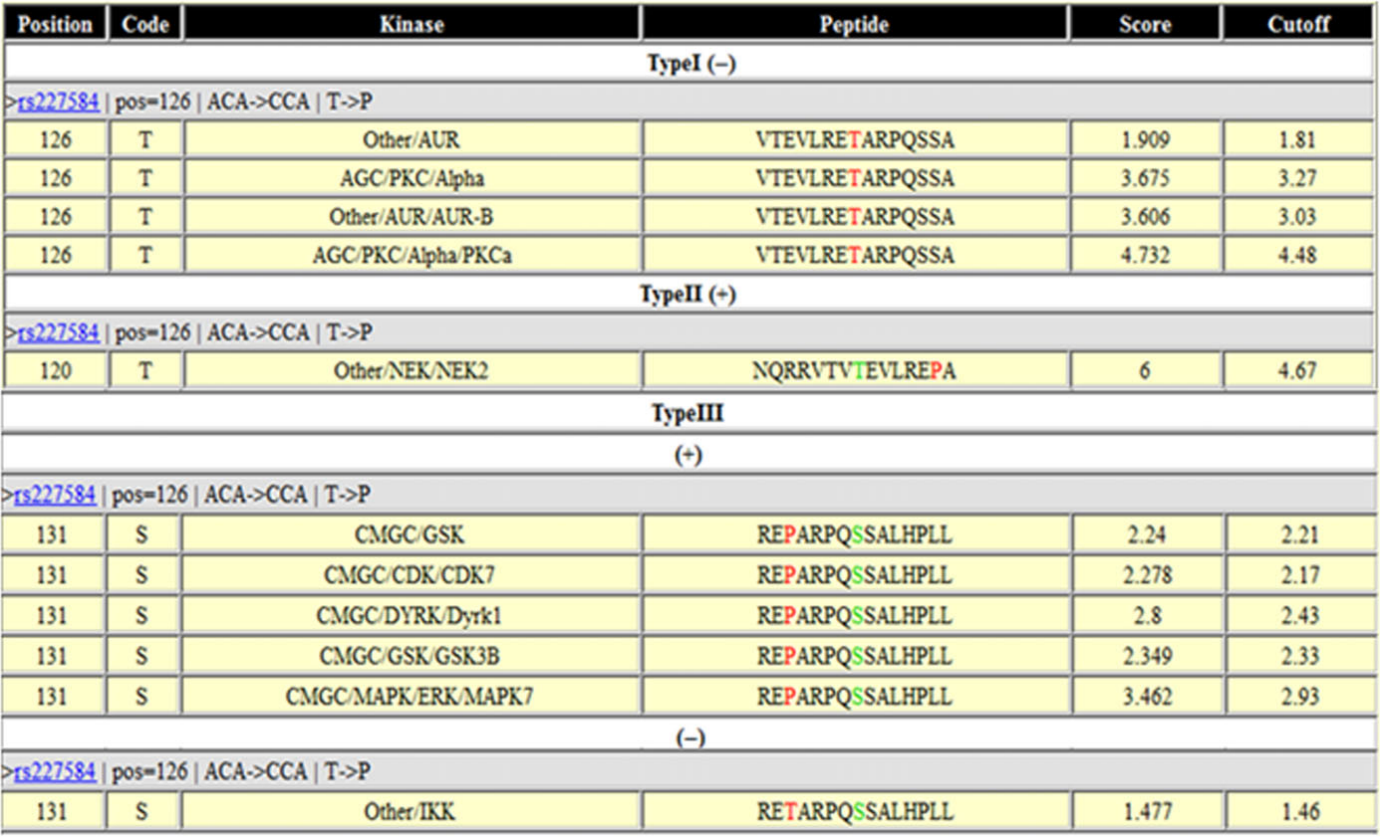
Predictive effects of phosSNP rs227584 on protein functions by GPS 2.0 software Type I (−) means an nsSNP that removes the phosphorylation site; Type II (+) means an nsSNP that creates one or multiple adjacent phosphorylation sites; Type III, an nsSNP that induces changes of protein kinase type(s) at adjacent phosphorylation sites

The potential impacts of the phosSNP rs227584 on protein phosphorylation were predicted and summarized in Figure 1. In general, the allele‐specific sequences encode different protein isoforms, which could change (create or abolish) the phosphorylation site(s) of the encoded protein product or change the kinase that catalysed the phosphorylation of target site(s).

Taking rs227584 into consideration, the *C17orf53* gene harbouring wild‐type allele C and mutant allele A encodes two different *C17orf53* protein isoforms with amino acid residue P and T at position 126 (abbreviated as *C17orf53*‐P126 and *C17orf53*‐T126), respectively. Bioinformatics prediction using GSP2.0 showed that, compared with mutant *C17orf53*‐T126, the wild‐type *C17orf53*‐P126 loses a phosphorylation site at amino acid 126 due to the residue substitution T126P, but makes an upstream residue T120 phosphorylatable. In another words, a new phosphorylation site (T120) was created while a nearby one at residue 126 was abolished. *NEK2* kinase was predicted, with the highest score of 6, to specifically catalyse the T120 phosphorylation in the wild‐type protein *C17orf53*‐P126. The substrate‐kinase relationship between *C17orf53* protein and *NEK2* kinase and the effect of rs227584 on *C17orf53* protein phosphorylation (T120) had not been experimentally validated before.

### 3.3 Experimental validation of rs227584 on protein functions

#### 3.3.1 Substrate‐kinase interaction

**Figure 2:**
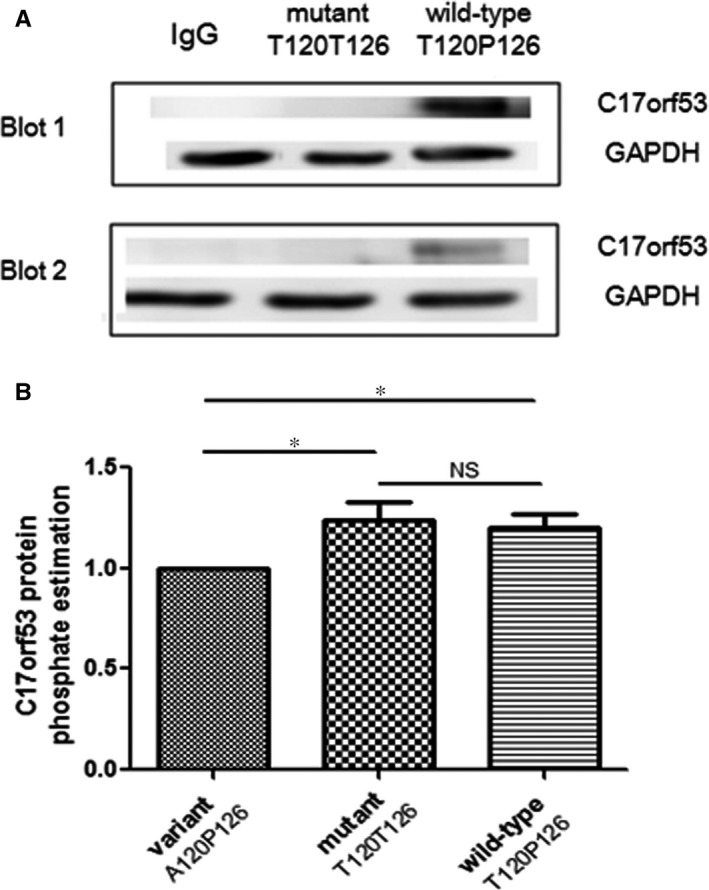
Effects of phosSNP rs227584 on protein phosphorylation (A) substrate‐kinase interaction assay for *C17orf53* and *NEK2* proteins. Two representative Western blots were presented. Samples were Co‐IP products of cell lysates from MG63 cells which was transiently transfected with variant, mutant, or wild‐type *C17orf53* genes, respectively. The Co‐IP product was prepared with anti‐*NEK2* antibody. (B) Allele‐specific effects of rs227584 on *C17orf53* total protein phosphorylation level. Presented are estimated phosphate levels in the immunoaffinity‐purified *C17orf53* proteins (mean and SD). The phosphate level under the condition of variant type *C17orf53* transfection (A120P126) was scaled as 1.0. *P < 0.05, as compared with the variant type. NS: insignificant

Our data indicated that the portion of wild‐type protein *C17orf53* can be pulled down together with kinase *NEK2* by anti‐*NEK2* antibody during Co‐IP process, as visualized on Western blot using anti‐*C17orf53* antibody (Figure 2A). In contrast, no mutant protein *C17orf53* was pulled down by anti‐*NEK2* antibody or visualized. The data displayed significant difference in interaction with kinase *NEK2* between wild‐type and mutant *C17orf53* protein in MG63 cells, suggesting allele‐specific substrate‐kinase interaction and impaired interaction between mutant *C17orf53* protein and *NEK2* kinase.

#### 3.3.2 Phosphorylation at C17orf53‐T120 affected by P126T substitution

Two‐sided student's t‐test showed that *C17orf53* protein phosphorylation level was significantly reduced in MG63 cells transfected with variant *C17orf53*‐**A120**P126, compared with that transfected with wild‐type *C17orf53*‐**T120**P126 (*P* = 0.008) or mutant *C17orf53*‐T120**T126** (*P* = 0.013) (Figure 2B). Nevertheless, no difference in protein phosphorylation level was detected between the wild‐type and mutant *C17orf53*. The data suggested that the SNP rs227584 does not significantly change the total phosphorylation level of protein *C17orf53*. With the presence of P126, the residue T120 was significantly phosphorylated, though the T120 phosphorylation was quantitatively offset by the loss of phosphorylation at amino acid site 126 due to the amino acid substitution from phosphorylatable T126 to nonphosphorylatable P126. The experimental data were consistent with the predicted effect of rs227584 on *C17orf53* protein phosphorylation at sites 120 and 126(Figure 1).

The data imply that *C17orf53* total protein phosphorylation is regulated by both amino acid substitutions P126T and T120A. It seems that the sacrifice of phosphorylation site T126 in wild‐type *C17orf53* protein was compensated for the gain of phosphorylation at nearby phosphorylation site T120. This sheds light on the phenomenon that there is no statistical difference in *C17orf53* total protein phosphorylation level between the wild‐type and mutant proteins, while both protein isoforms maintain higher phosphorylation levels than the variant *C17orf53*.

### 3.4 Effects of rs227584 on osteoblastogenesis in vitro

#### 3.4.1 C17orf53 expression in stably transfected MG63 cells

**Figure 3:**
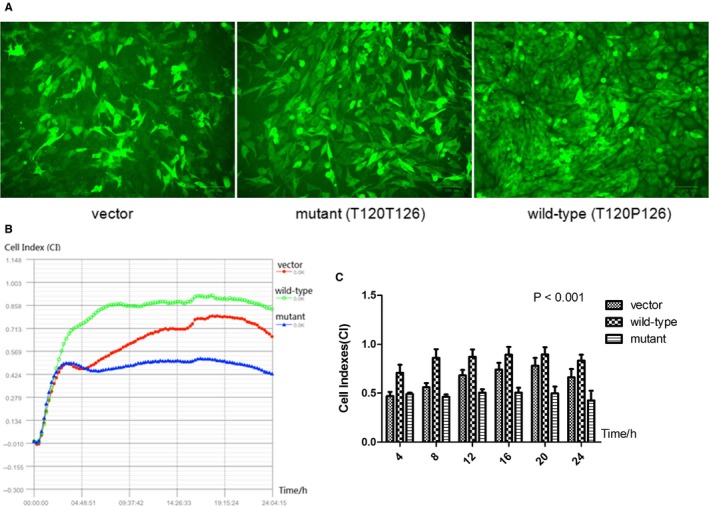
Allele‐specific effects of rs227584 on the growth of stably transfected MG63 cells. Cell culture images with fluorescence microscope. Presented are representative images for three types of stably transfected MG63 cells. A, 24‐h growth curve. Presented are real‐time cell indexes (CI) recorded at every 10‐min within 24 h. B, MG63 growth in vitro recorded at multiple time‐points. Presented are cell indexes (CI) monitored for 24 h in cell growth assay. The data are described as mean ± SD

Cell culture imaging indicated that MG63 cells were infected successfully with high efficiency (Figure 3A). Besides, quantitative real‐time PCR revealed that the *C17orf53* mRNA expression was significantly up‐regulated in MG63 cells transfected with *C17orf53* gene vs empty vector (Figure S2), highlighting the overexpression of both wild‐type and mutant *C17orf53* in MG63 cells.

#### 3.4.2 Effect of rs227584 on cell growth

Dynamic monitoring of cell growth for 24 hours showed that, transfection with wild‐type *C17orf53*‐T120P126 obviously promoted MG63 cell growth in contrast to the negative control. In contrast, the cell growth was obviously inhibited by mutant *C17orf53*‐T120T126 (Figure 3B). MG63 growth in vitro recorded at multiple time‐points (Figure 3C) showed that the growth differences were significant (*P *< 0.001). The data indicated allele‐specific effect of rs227584 on osteoblastic growth.

#### 3.4.3 Effect of rs227584 on expression of osteoblastic marker genes

**Figure 4:**
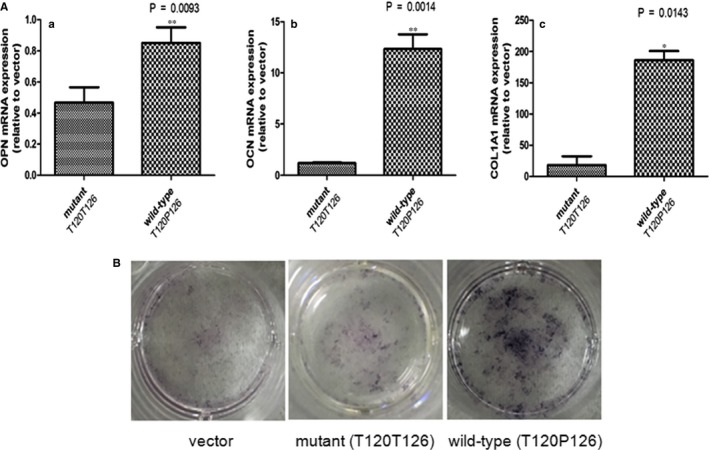
Allele‐specific effects of rs227584 on osteoblastogenesis of stably transfected MG63 cells. A, Allele‐specific effects of rs227584 on osteoblastic gene expression (*OPN*,*OCN,* and *COL1A1*) in MG63 cells after 72‐h culture. Presented are mRNA abundances in MG63 cells. Raw data were normalized against the internal control GAPDH mRNA level (mean and SD). Negative control refers to empty vector transfection.*P < 0.05; **P < 0.01, as compared with mutant *C17orf53* transfection (T120T126). B, Allele‐specific effects of rs227584 on the osteoblastic *ALP* activity in MG63 cells after 7‐day culture

Quantitative real‐time PCR experiments revealed that the MG63 cells transfected with wild‐type *C17orf53*‐T120**P126** presented significantly higher mRNA expression of osteoblastic genes (*OPN, OCN, COL1A1*) compared with cells transfected with mutant *C17orf53*‐T120**T126** (Figure 4A).

#### 3.4.4 Effect of rs227584 on osteoblast activity

*ALP* staining assay showed that transfection with wild‐type *C17orf53*‐T120P126 significantly increased *ALP* enzyme activity in MG63 cells, as compared with the negative control (Figure 4B). In contrast, the stimulatory effect on *ALP* activity was significantly reduced by mutant *C17orf53*‐T120T126, as compared with the wild‐type (Figure 4B). The data indicated allele‐specific effect of rs227584 on osteoblastic differentiation and activity.

### 3.5 eQTL effect of phosSNP rs227584 from phenotype‐genotype integrator

There is only one record archived in Phenotype‐Genotype Integrator by Dec 6, 2017, supporting the eQTL effect of SNP rs227584 (Table 1). Specifically, SNP rs227584 exerts trans effect on KLF10 gene and cis effect on G6PC3, ASB16‐AS1 (C17orf65, 13.5 kb downstream of *C17orf53*), and SLC4A1 genes. A recent eQTL study on whole blood cells in a large cohort reported its cis effect on the above three genes (Table 1), as well as the ASB16 gene,^20^ which is partially overlapping with the ASB16‐AS1 gene in DNA sequence. Besides, it was reported that the mutant allele A at rs227584 correlated with increased C17orf65 expression in monocytes, adipose tissue, whole blood, and lymphoblasts.^21^ The above data supported that phosSNP rs227584 plays a significant role in regulating gene expression in multiple other types of human cells, including the osteoclast precursor monocytes.

**Table 1:** eQTL effects of phosSNP rs227584 archived in phenotype‐genotype integrator and in human whole blood cells^20^

SNP_ID	Gene symbol^a^	Gene description	Chr	Is Cis	QTL ID	RSq^b^	Log10P^c^
rs227584	C17orf65(ASB16‐AS1)	ASB16 antisense RNA 1	17	1	956227	0.003	−4.2
rs227584	SLC4A1	Solute carrier family 4 member 1	17	1	1657503	0.027	−32.1
rs227584	G6PC3	Glucose‐6‐phosphatase catalytic subunit 3	17	1	3881240	0.012	−14.1
rs227584	KLF10	Kruppel‐like factor 10	8	0	17336955	—	—

a GeneSymbol: Gene Symbol for the Transcript Cluster ID.

b RSq: Rsq for the SNP—Transcript Cluster pair.

c log10P: Log10 *P*‐value for the SNP—Transcript Cluster pair.

## 4 DISCUSSION

This study represents our first endeavour to illustrate the molecular and cellular mechanism underlying the association of a key SNP, that is, rs227584, with bone phenotypes (BMD/fracture risk) in humans. Through in‐depth functional studies in vitro, we characterized and validated that the SNP rs227584 changes substrate‐kinase interaction between protein *C17orf53* and kinase *NEK2* and affects *C17orf53* protein phosphorylation sites in bone cells, regulates osteoblastic gene expression, and influences osteoblast growth and osteoblastic differentiation and activity. The present findings explained the prior association observed in large human populations, and warranted the significance of SNP rs227584 to bone biology.

PhosSNP rs227584 is located in *C17orf53* gene. *C17orf53* (Chromosome 17 open reading frame 53) is a protein‐encoding gene, which is conserved in mouse, chicken, lizard, and zebrafish. It was expressed in diverse tissues in human body, like brain, colon, fat, kidney, and so on, but most abundantly in bone marrow and testicles (https://www.ncbi.nlm.nih.gov/pubmed). In regard to the *C17orf53* protein, there was relatively limited understanding about its molecular and cellular functions and biological effects. Nonetheless, association of rs227584 with BMD variation has been suggested in previous meta‐analysis.^21^ A large‐scale study involving 15,375 individuals of European and Oceanian ancestry revealed that rs227584 was associated with both hip BMD (*P *< 0.001) and spine BMD(*P* = 0.058).^22^ To be noted, the observed effect on BMD in human populations was consistent with its effects on osteoblastic functions observed in MG63 in the present study. Concretely, carriers of risk allele A has significantly inhibited osteoblast growth and decreased osteoblastic activity and be associated with significantly lower BMD than carriers of the alternative allele C.

The quantitative real‐time PCR data showed that *C17orf53*‐T126 (risk allele A at rs227584) transfection significantly decreased the expression of osteoblastic‐related marker genes (*OPN, OCN, COL1A1*) and attenuated the growth and differentiation of osteoblast in cell culture, in contrast to *C17orf53*‐P126 (allele C at rs227584). Our findings implied that rs227584 affected osteoblastogenesis via regulating the osteoblastic gene expression. OF is heritable by mechanisms that are partly independent of BMD,^23^ but to some extent, BMD and fracture were genetically correlate with each other.^24^, ^25^, ^26^ In a study including 8594 cases and 23218 controls, it revealed that rs227584 harbouring allele A was associated with increasing risk of any fracture type.^21^ The data highlighted that rs227584 was not only associated with BMD variation but also correlated with fracture risk. To the best of our knowledge, our present work for the first time dissected and characterized the molecular functions of SNP rs227584 which significantly affects the growth, differentiation, and activity of bone formation cells.

Our present work indicated that the risk allele A at SNP rs227584 does not significantly change the overall phosphorylation level of *C17orf53* protein. Instead, it could change *C17orf53* protein interaction with kinase *NEK2* and change the target site of phosphorylation to be T126 instead of T120. Specifically, the interaction with *NEK2* was present with wild‐type *C17orf53*‐T120P126 but absent with mutant *C17orf53*‐T120T126. In the presence of P126, the overall *C17orf53* protein phosphorylation level was significantly changed by the residue substitution T120A, indicating that the amino acid residue T120 is a target site of phosphorylation, which is catalysed by kinase *NEK2*. The observation of no significant difference in overall phosphorylation level between wild‐type and mutant *C17orf53* protein isoforms coincides with the bioinformatics prediction, supporting that the creation of a new phosphorylation site at residue 120 accompanies with the removal of a nearby one at residue 126.

In this study, we found that wild‐type *C17orf53*‐T120P126 significantly promoted MG63 cell growth compared to the mutant type. Furthermore, only the wild‐type but not the mutant *C17orf53* protein could interact with *NEK2* kinase. Coincidently, *NEK2* kinase is an important protein involved in mitotic process and cell cycle regulation.^27^ This protein is located at the centrosome, which accumulates progressively throughout the S phase and reaches maximal levels in late G2 phase. It revealed that *NEK2* shows constitutive catalytic activity and phosphorylates proteins involved in centrosome duplication.^28^ Among its related pathways are “Regulation of *PLK1* Activity at G2/M Transition” and “*APC‐Cdc20* mediated degradation of *Nek2A*” (https://genecards.com/). We infer that the *NEK2* kinase may play an important role in mediating the effects of rs227584 on osteoblast growth, and similar molecular pathways might be involved.

Over the past decades, extensive genetic and genomic studies have conducted to discover genes/SNPs associated with BMD variation in human populations.^29^, ^30^, ^31^, ^32^, ^33^, ^34^ However, our understanding about the underlying association mechanism is very limited. Besides, a majority of phosSNPs in the GPS 2.0 database were predicted, and their potential effects on protein phosphorylation have not been validated by experiments.^35^ From the perspective of protein phosphorylation, gene expression, and cellular functions, the present study represents our pursuant efforts to ascertain the functions of a key phosSNP, rs227584 in bone cell, so as to fill the gap of knowledge about its link with osteoporosis risk in human populations of multiple ancestries.

**Figure 5:**
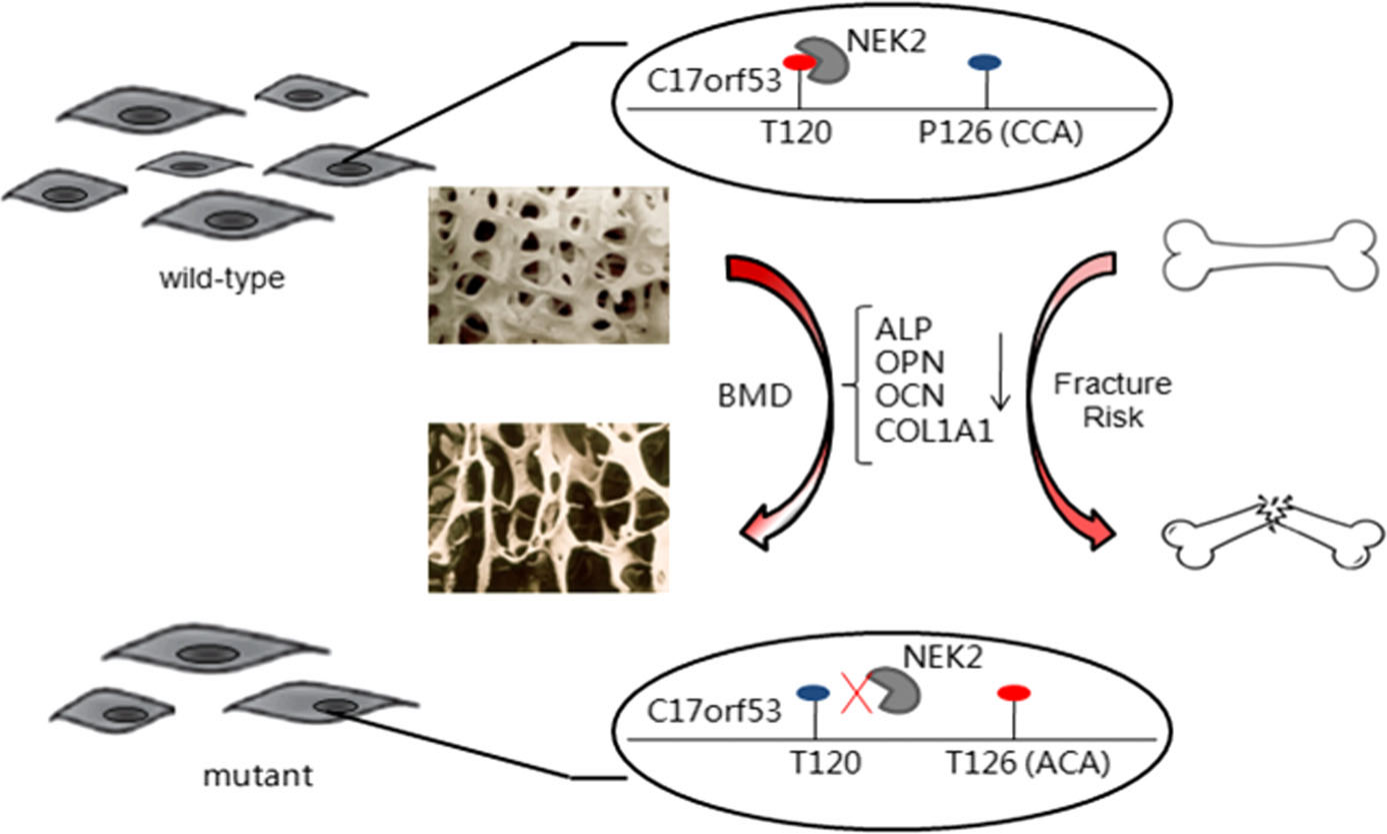
A schematic diagram illustrating the biological and clinical effects of the SNP rs227584 on bone. 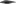 osteoblast; 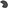 kinase *NEK2*; 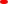 phosphorylated residue; 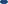 unphosphorylated residue

In summary, this study was focused on phosSNPs significant for human BMD and fracture risk and characterized the functions of a representative phosSNP of interest through in vitro molecular and cellular studies. Based on the collected evidences, we proposed that the SNP rs227584, via switching protein‐kinase interaction and probably altering *C17orf53* protein phosphorylation site, significantly regulates human osteoblastic gene expression, and influences osteoblast growth and osteoblastic activity, hence to affect BMD and fracture risk in humans (Figure 5). The present study highlights the significance of single‐nucleotide or amino acid polymorphism for protein molecular functions and cellular activity, which may shed new light on phosSNP‐involved molecular pathogenesis of human diseases.

## CONFLICT OF INTEREST

The authors confirm that there are no conflicts of interest.
