# Supplementary figures and images for: Why SNP rs227584 is associated with human BMD and fracture risk? A molecular and cellular study in bone cells
Source: J Cell Mol Med. 2018 Oct 28;23(2):898–907. doi: 10.1111/jcmm.13991 (PMC6349212; doi:10.1111/jcmm.13991)

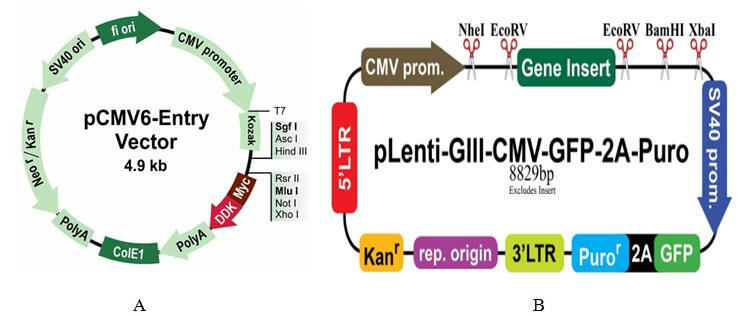

Supplement: Supplementary file 1 [file JCMM-23-898-s001.tif]

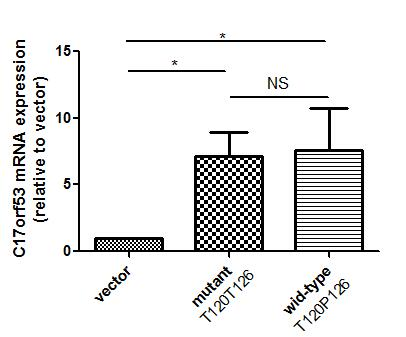

Supplement: Supplementary file 2 [file JCMM-23-898-s002.tif]
