# Supplementary material for: Why SNP rs227584 is associated with human BMD and fracture risk? A molecular and cellular study in bone cells
Source: J Cell Mol Med. 2018 Oct 28;23(2):898–907. doi: 10.1111/jcmm.13991 (PMC6349212; doi:10.1111/jcmm.13991)
Supplement: Supplementary file 3 [file JCMM-23-898-s003.docx]

**Supplemental Table 1 Sequences for Primer/Probe Sets Used in qPCR**

| Primer | Sequences 5’ to 3’ |
| --- | --- |
| OPN-F | CCATACCAGTTAAACAGGCTG |
| OPN-R | TCAGGGTTTAGCCATGTGG |
| OCN-F | GAGAGCCCTCACACTCCTCG |
| OCN-R | GTCTCTTCACTACCTCGCTGCC |
| COL1A1-F | ACCGCCCTCCTGACGC |
| COL1A1-R | CGTTGTCGCAGACGCAGAT |
| GAPDH-F | TTCTTTGCAGCAATGCCTC |
| GAPDH-R | GACTGTGGTCATGAGTCCT |
