# Supplementary material for: Why SNP rs227584 is associated with human BMD and fracture risk? A molecular and cellular study in bone cells
Source: J Cell Mol Med. 2018 Oct 28;23(2):898–907. doi: 10.1111/jcmm.13991 (PMC6349212; doi:10.1111/jcmm.13991)
Supplement: Supplementary file 4 [file JCMM-23-898-s004.docx]

**Supplemental Table 2 PhosSNPs Significant for Bone Density and Fracture Risk Archived in NHGRI and dbGap Databases**

| PhosSNP ID | WT^e^/MUT^f^ alleles | Gene Symbol | AA^g^ change | PhosSNP type^a^[[6](#_ENREF_6)] | Trait | Reference  (PubMed ID) |
| --- | --- | --- | --- | --- | --- | --- |
| rs227584 | C/A | C17orf53 | P126T | TypeⅠ(-)^b^; TypeⅡ(+)^c^ ;TypeⅢ(+)^d^; TypeⅢ(-)^d^ | Bone Density; Fracture, Bone | 22504420 |
| rs3736228 | C/T | LRP5 | A1330V | TypeⅢ(+)^d^; TypeⅢ(-)^d^ | Bone Density | 22504420; 18455228 |
| rs3755955 | G/A | IDUA | R105Q | TypeⅡ(+)^c^; TypeⅢ(-)^d^ | Bone Density | 22504420 |
| rs10416265 | A/G | GPATCH1 | H724R | TypeⅢ(+)^d^; TypeⅢ(-)^d^ | Bone Density; Fracture,Bone | 24430505 |
| rs1054627 | G/A | IBSP | G195E | TypeⅢ(+)^d^; TypeⅢ(-)^d^ | Bone Density | 21533022 |

^a^ The types of phosSNPs are defined as follows (predicted by GPS2.0 software):

^b^ TypeⅠ(-), an nsSNP that removes the phosphorylation site;

^c^ TypeⅡ(+),an nsSNP that creates one or multiple adjacent phosphorylation sites;

^d^ Type Ⅲ, an nsSNP that induces changes of protein kinase type(s) at adjacent phosphorylation sites;

^e^ WT= wild-type; ^f^ MUT= mutant; ^g^ AA= amino acid.
